# AI-Powered Personal Tutors as a Scalable Solution to the 2-Sigma Problem in Pharmacy Education

**DOI:** 10.3390/pharmacy14070151

**Published:** 2026-10-14

**Authors:** Maria Victoria Tejada-Simón

**Affiliations:** Department of Pharmacological and Pharmaceutical Sciences, College of Pharmacy, University of Houston, Houston, TX 77004, USA; mvtejada@central.uh.edu

**Keywords:** artificial intelligence, mastery learning, pharmacy education, foundational sciences, engagement, student-centered learning

## Abstract

**Background:** The “2 Sigma Problem”, where individualized tutoring outperforms conventional instruction by two standard deviations, remains a persistent challenge in large-enrollment pharmacy courses. Generative AI tutors, constrained to instructor-verified content, offer a scalable, equitable approximation of personalized support. **Methods**: Four course-specific AI tutors were deployed across first-year (P1; *n* = 108–119/cohort) and third-year (P3; *n* = 98–108/cohort) Doctor of Pharmacy cohorts (Fall 2024–Spring 2026) using ChatGPT-4o and PlayLab with retrieval-augmented generation. Effectiveness was evaluated through longitudinal grade analysis, American Association of Colleges of Pharmacy proficiency outcomes, satisfaction surveys, and anonymized interaction log analysis. **Results**: Engagement was heterogeneous across curricular formats: 15-week P1 courses supported sustained weekly usage for foundational concept reinforcement, whereas 4-week P3 modules exhibited lower, targeted utilization focused on rapid self-assessment. In P1 Biochemistry, tutor integration was associated with longitudinal cohort exam improvements. The overall targeted metabolism exam mean rose from 74.0% (Fall 2023, no AI) to 81.4% (Fall 2024, AI tutor) and 83.6% (Fall 2025, AI tutor), with active users (*n* = 16, 85%, Fall 2024) outperforming non-users (*n* = 16, 79.3%, Fall 2024) by 5.7 percentage points, and with higher mean scores observed across progressively higher usage tiers. AACP proficiency means increased from 77.9% to 82.1%, exceeding the 80% benchmark. In P1 Physiology and P3 modules, cohort performance remained consistently high (83.7–88.2%), maintaining AACP benchmark proficiency across all observation cycles. Student perceptions were positive regarding conceptual clarification and adaptive explanations. **Conclusions**: Curriculum-aligned AI tutors represent a viable strategy for approximating individualized instruction in pharmacy courses. Course-specific AI tutors demonstrate feasibility across PharmD curricula, functioning as continuous retrieval-practice tools in foundational sciences and as targeted self-assessment aids in accelerated modules. Educational benefits and adoption dynamics are moderated by curricular pace, subject complexity, and assessment structures, highlighting key considerations for broader health sciences implementation.

## 1. Introduction

The “2 Sigma Problem,” identified by the educational psychologist Benjamin Bloom, suggests that the average student tutored one-to-one using mastery learning techniques performs significantly better than 98% of students in a traditional classroom setting [1]. However, scaling this level of personalization in large pharmacy cohorts has historically been an impossible task, especially in classrooms of 100 or more. While 1:1 tutoring is the gold standard for developing mastery, it is logistically and financially impossible to scale in modern higher education [1,2,3], particularly in demanding disciplines like the health professions [4,5], where large class sizes often prevent individualized interaction between instructors and students. Instructors, in all educational settings, have observed this challenge firsthand, vocalizing the frustration and the inability to reach every student in a one-on-one format to answer questions and conduct reviews in their classrooms [5,6,7,8,9].

However, recent advancements in Generative Pre-trained Transformers (GPTs), as large language models built on the transformer architecture [10] and trained on massive text datasets to generate human-like responses [11], provide a potential answer to this decades-old problem. By mimicking the individualized guidance of a human tutor, AI-powered assistants can provide immediate, 24/7 feedback and adaptive explanations to specific content [12,13]. While GPT-based tutoring has shown promise in K-12 settings (primary and secondary education, kindergarten through 12th grade; [14,15]) and as virtual assistants in non-educational contexts [16], applications in health profession higher education remain largely limited [17,18], and evidence of educational outcomes is poor [19]. Despite growing interest, barriers persist (such as a lack of familiarity with AI and unclear roles in pharmacy education; [17]), leaving applications in health professions higher education largely unexplored.

This manuscript details the development of these types of AI tutors and explores their impact on student engagement and performance in our PharmD curriculum program (*Doctor of Pharmacy, a four-year professional doctorate program in the USA*), aiming to determine whether AI can effectively “crack the 2-sigma code” in a large classroom environment. Our approach moves beyond traditional “one-size-fits-all” instruction by leveraging Generative AI to create a “digital mentor/tutor”. Crucially, this innovation addresses the needs of students by using a variant of RAG technology (Retrieval-Augmented Generation, an AI architecture that grounds language models in instructor-curated course materials to reduce hallucinations [20]) to constrain AI responses to specific course transcripts and videos, ensuring accuracy and curriculum alignment [21]. Additionally, by developing these tools and allowing students to interact with AI early in their careers, we also support professional development, a recognized priority as pharmacists must develop AI literacy and digital competencies to navigate technology-driven healthcare environments [22,23,24,25]. This early exposure prepares students for a profession where AI literacy is increasingly essential [26,27] while also supporting time management and continuous improvement, thereby “humanizing” the educator’s role by offloading administrative burdens.

While generative AI presents substantial opportunities to approximate Bloom’s 2 Sigma model of individualized tutoring at scale, empirical evidence regarding multi-course implementation, longitudinal academic outcomes, and curricular moderation in health sciences education remains sparse. Therefore, the primary objective of this study was to design, deploy, and evaluate four course-specific generative AI tutors grounded in instructor-curated content across foundational (first-year, P1) and clinical (third-year, P3) Doctor of Pharmacy cohorts over a two-year period (Fall 2024–Spring 2026). Specifically, this investigation aimed to: (1) evaluate the association between AI tutor engagement and student academic performance on modular course examinations and American Association of Colleges of Pharmacy (AACP) standardized proficiency benchmarks; (2) characterize student adoption and utilization patterns across distinct curricular structures (16-week semester-long courses vs. compressed 4-week clinical modules); and (3) assess student satisfaction, perceived utility, and implementation barriers across platform transitions.

## 2. Materials and Methods

### 2.1. Study Design and Participants

This study was conducted at the University of Houston—College of Pharmacy (UH-COP), with first- and third-year students in a Doctor of Pharmacy program. Each class had 100+ students. Approval was obtained from the Institutional Review Board (IRB) at the University of Houston (STUDY00005098, approval letter 10 March 2025).

Participants included students enrolled in the required first-year Biochemistry or Physiology I course offered during the Fall semester, a third-year Neurology module offered during the Fall semester, as well as a third-year Psychiatry module offered during the Spring semester. This study was developed and completed with students enrolled for Fall 2024, Spring 2025, Fall 2025, and Spring 2026.

A dual informed consent procedure was implemented in accordance with institutional human subject guidelines:

- A waiver of formal signed consent was granted by the IRB for the analysis of de-identified, aggregate course examination averages, historical cohort comparisons (Fall 2023 baseline), and anonymized platform-level interaction server logs, as these represented standard educational evaluation of non-mandatory learning resources.
- For student perception surveys and the pilot linkage analysis in Fall 2024, electronic informed consent was obtained. Students were informed of the study objectives, the voluntary nature of participation, data confidentiality protections, and that non-participation or withdrawal would carry no penalty or effect on course grades. For the Fall 2024 pilot subgroup (*n* = 32), students provided explicit authorization permitting researchers to cross-reference their self-reported usage tiers with examination records using de-identified codes.

### 2.2. Course Description and Multi-Tiered AI Tutoring Instructional Intervention

*AI Tutor Design and Platform Deployment***.** Four distinct AI tutors were deployed across the first-year foundational (P1) and third-year clinical (P3) pharmacy courses. A comprehensive summary of course structures, cohort enrollments, intervention durations, and targeted assessment milestones is presented in Table 1 and Table 2, with technical tutor specifications and survey responses (students who completed the voluntary evaluation survey, regardless of their tutor usage status) detailed in Table 2.

Tutors were grounded in instructor-verified lecture transcripts, slides, and learning objectives using retrieval-augmented generation (RAG, [20]) principles. System prompts were configured to encourage Socratic dialogue, prompting students to articulate their reasoning, and to establish domain boundaries restricting responses to course materials.

Initial implementation in Fall 2024 utilized OpenAI’s ChatGPT platform. Beginning in Spring 2025, deployments transitioned to PlayLab (PlayLab Education Inc.). This migration addressed key institutional needs: eliminating student subscription requirements, standardizing access without third-party account friction, and enabling centralized collection of de-identified interaction metrics. Complete system protocol and detailed technical platform comparisons are provided in the Supplementary Materials. The bots’ design ensured the tools functioned as foundational science tutors rather than clinical decision support systems, respecting the expertise of clinical instructors while providing 24/7 academic support.

To facilitate a meaningful comparison of student performance pre- and post-intervention with AI tutor implementation, the Fall 2023 cohort was designated as the primary control group. Earlier cohorts (Fall 2020–2022) were excluded from comparative analyses due to significant instructional disruptions caused by the COVID-19 pandemic. Specifically, the Fall 2020 and Fall 2021 cohorts received fully online instruction, while the Fall 2022 cohort utilized a hybrid format. These delivery modalities introduced confounding variables, including differential access to synchronous instruction, altered assessment formats, and modified peer learning environments, which precluded valid comparison with subsequent in-person cohorts. Consequently, Fall 2023 was selected as the baseline, as it marked the return to normative, fully in-person instruction, providing a methodologically defensible foundation for evaluating the impact of AI tutor integration in academic years 2024 and 2025 (Table 3).

### 2.3. Data Collection and Analysis

A mixed-methods approach was employed to evaluate the effectiveness and student perception of the AI tutors across all cohorts.

- *Qualitative data and log analysis*: Qualitative data were derived from open-ended survey responses and, when available, anonymized interaction logs. Open-ended survey responses were analyzed inductively to identify recurring themes regarding perceived benefits, barriers to adoption (as the students who actively engaged with the AI tutor platform), and suggestions for iterative improvement.
  Regarding interaction log analysis, for PlayLab-based deployments, anonymous interaction logs were reviewed through the administrative dashboard to characterize student query types, recurring misconceptions, and the tutor’s pedagogical scaffolding strategies, such as stepwise explanations, concept integration, self-testing, and follow-up prompts. For the Fall 2024 ChatGPT deployment, log analysis was limited to URL links voluntarily shared by students via anonymous Google Forms, as the platform did not support centralized administrative access at the time of the pilot. Recurrent themes were also identified.
  Thematic coding of the surveys was conducted by the instructor-researcher. Given the pilot nature of the study, a formal inter-rater reliability assessment was not performed, but it represents a priority for future investigations.
- *Quantitative assessment*: Student satisfaction and perceived effectiveness of each AI tutor were assessed using a purpose-designed Likert-scale questionnaire administered via Qualtrics at the end of each semester. The questionnaire evaluated three primary dimensions: (1) accessibility and usability, (2) clarity and quality of explanations, and (3) perceived impact on learning and engagement. Items were rated on a five-point scale ranging from Strongly Agree (5) to Strongly Disagree (1). Tutor usage frequency was also captured by a categorical response scale (0–2—low, 3–5—medium, 6–8—high, or more than 7 sessions—very high).

For the Fall 2024 Biochemistry cohort (Dr. T’s Tutor, ChatGPT), a survey instrument was administered to a small group of both active users (*n* = 16) and a comparison group of non-users (*n* = 16), representing a 13.4% adoption rate of the classroom enrollment. This pilot assessment evaluated the platform’s ease of use, the clarity of biochemical explanations, alongside student perceptions of improved understanding, the relevance of provided examples, and the effectiveness of analogies.

For the Fall 2025 Biochemistry and Physiology cohort (P1), a similar questionnaire was administered to the active users (*n* = 23; 20.9% response rate). The version included items assessing the tutor’s ability to provide relevant examples that clarified difficult topics, enjoyment of interaction during study sessions, perceived personalization and adaptability of responses, generation of assessment-style questions, and usage frequency.

For the Fall 2025 Neurology cohort and Spring 2025 and 2026 Psychiatry cohorts, a parallel survey was administered to evaluate these tutors.

Given the pedagogical differences among disciplines, outcome measures were specifically mapped to each tutor’s modular focus and the corresponding faculty instructional assignments. This also allowed us to maintain consistency with the longitudinal grade trend analysis and facilitate direct comparison with historical pre-intervention cohort data. Academic performance outcomes are reported using the following course-specific measures:

- P1 Biochemistry: Exam 2 was designated as the primary milestone because Dr. T’s Tutor was specifically trained on carbohydrate, lipid, and amino acid metabolism, which constitutes ~90% of Exam 2 content.
- P1 Physiology: Exam 4 was evaluated because ANSwer Bot was designed to support the autonomic nervous system and neuromuscular physiology units assessed on this specific examination.
- P3 Clinical Modules (Neurology & Psychiatry)**:** Because pathophysiology and pharmacology content was distributed across the entire 4-week block, overall course grade averages served as the summative outcome.

This targeted outcome alignment ensures that academic performance measurements reflect student learning on the exact curriculum supported by each AI tool, avoiding contamination from unrelated course modules.

For the subgroup analysis comparing active tutor users and non-users within the Fall 2024 Biochemistry cohort, academic performance was additionally operationalized using only the examination items directly aligned with Dr. T’s Tutor’s curated content domain, specifically, metabolism-related questions comprising approximately 90% of the total items on Exam 2. This construct-aligned scoring approach was intentionally selected to maximize internal validity by isolating student performance in the precise curricular domain in which AI-assisted learning was available, thereby reducing noise introduced by examination items covering content outside the tutor’s knowledge base. Both overall Exam 2 scores and metabolism-aligned scores are reported for the Fall 2024 Biochemistry cohort to allow direct comparison across analytical levels.

The American Association of Colleges of Pharmacy (AACP) provides standardized competency domains used for curricular quality assessment, and the 80% threshold serves as a widely recognized benchmark for demonstrated subject mastery. Thus, to evaluate the longitudinal impact of AI tutor integration on specific learning outcomes, student performance data from Fall 2023 to Spring 2026 were mapped to the AACP standardized proficiency framework.

For biochemistry, this analysis tracked eleven distinct proficiency domains (43001-01 through 4300-11) with a targeted evaluation of the three domains directly aligned with the AI tutor’s curated knowledge base: carbohydrate/amino acid/lipid metabolism (4300-05), fed states and diabetes (4300-06), and vitamins and minerals (4300-08).

For Physiology, our analysis tracked seven distinct proficiency domains (4320-01 through 4320-07), with a targeted evaluation of the one domain directly aligned with the anatomy and physiology of the autonomic nervous system and visceral reflexes.

For Neurology, seven proficiency domains were tracked (5335-01 through 5335-07) with a targeted evaluation of the two domains directly aligned with the pharmacology and pathophysiology of pain and anesthesia: 5335-01 Apply the fundamental concepts of pathophysiology to the overall disease process and clinical decision-making for neurological diseases; and 5335-03 Apply the fundamentals of pharmacology and toxicology to the therapeutic clinical decision-making for neurological diseases.

For Psychiatry, seven proficiency domains were tracked (5368-01 through 5368-07) with a targeted evaluation of the two domains directly aligned with the pharmacology and pathophysiology of schizophrenia, delirium, depression, and bipolar disorder: 5368-01 Apply the fundamental concepts of pathophysiology to the overall disease process and clinical decision-making for psychiatric diseases; and 5368-03 Apply the fundamentals of pharmacology and toxicology to the therapeutic clinical decision-making for psychiatric diseases.

Performance in these domains was benchmarked against a standardized 80% proficiency target to identify shifts across the pre-intervention (Fall 2023), ChatGPT (Fall 2024), and PlayLab (Fall 2025, Spring 2025, 2026) cohorts.

- *Statistical Analysis*: Quantitative data were analyzed descriptively, with positive response rates defined as the sum of strongly agree and agree responses. To examine the relationship between tutor engagement and academic outcomes, a Pearson correlation analysis was conducted to examine the relationship between usage frequency and exam performance.

## 3. Results

Based on the data collected, this results section is organized in a logical flow that moves from broad engagement patterns to specific academic outcomes and student perceptions.

**Figure 1:**
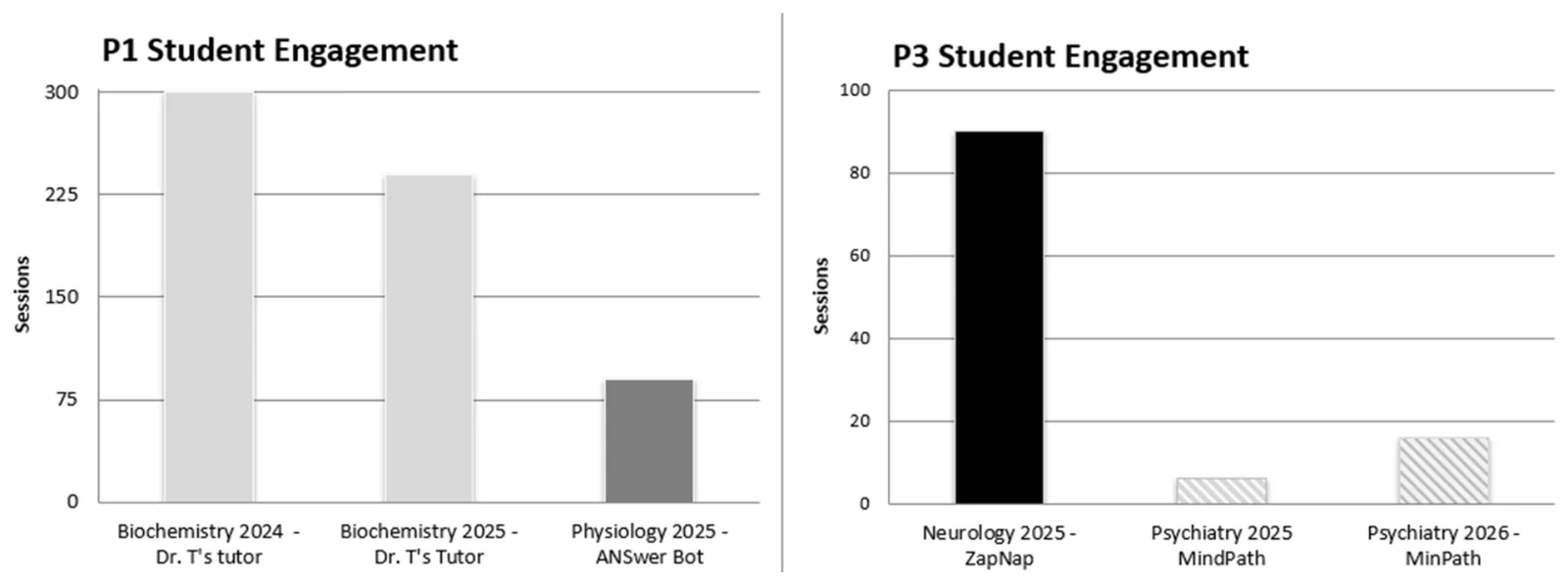
Descriptive summary of AI tutor interaction volume across course deployments (2024–2026). Bar height represents the total recorded student interaction sessions per course deployment. P1 foundational courses (15-week semester, *n* = 108–119) and P3 modules (4-week blocks, *n* = 98–108) represent fundamentally distinct curricular structures and usage intentions (foundational reinforcement vs. accelerated self-assessment). AI tutor platforms are distinguished by filled bars as depicted in the graph. Tutor names are indicated on the X-axis. Session counts reflect total anonymized interactions logged per deployment period. Data are presented descriptively to illustrate feasibility and adoption within each educational context rather than as an evaluative cross-cohort comparison. The substantial difference in session volume between P1 and P3 cohorts reflects differences in course duration rather than differences in student motivation or tutor quality.

1. Participant Flow and AI Tutor Engagement—Total platform engagement across all deployments is presented in Figure 1. Total engagement metrics were collected across six deployments in two distinct educational settings: 15-week foundational courses (P1) and accelerated 4-week pharmacotherapy modules (P3). Engagement patterns are reported descriptively to characterize implementation dynamics within each specific course structure, rather than as a direct head-to-head comparative outcome, given the non-equivalent course durations, learning objectives, and student usage intentions. Engagement patterns varied substantially between curricular levels, reflecting fundamental structural differences between the P1 and P3 learning environments.

Among first-year students, engagement with the AI tutors was notably high. Dr. T’s Tutor (Biochemistry, 2024; ChatGPT) recorded 300 access sessions, representing the highest engagement across all deployments. The revised Dr. T’s Tutor (Biochemistry, 2025; PlayLab) recorded 240 sessions, while Answer Bot (Physiology, 2025; PlayLab) recorded 90 sessions.

Adoption rates (as the students who actively engaged with the AI tutor platform) differed in their derivation across cohorts and should be interpreted accordingly. For the Fall 2024 ChatGPT pilot, the student adoption rate of at least 13.4% (*n* = 16 of 119 enrolled) reflects confirmed active users, as subgroup inclusion required documented tutor engagement. For the Fall 2025 PlayLab cohort, the 20.9% figure represents the voluntary survey response rate (*n* = 23 of *n* = 110 enrolled; as the students who completed the voluntary evaluation survey, regardless of their tutor usage status) rather than a verified measure of platform utilization. As PlayLab interaction logs capture all sessions regardless of survey participation, actual tutor adoption in this cohort likely exceeded the survey response-derived estimate; the 20.9% figure therefore represents a conservative lower bound.

Engagement among third-year students was markedly lower in absolute terms (Figure 1), though qualitatively distinct in nature. ZapNap (Neurology, 2025; PlayLab) recorded 90 sessions, while MindPath (Psychiatry, 2025) and MindPath (Psychiatry, 2026) recorded 6 and 16 sessions, respectively. When engagement was normalized for cohort size and instructional duration (Table 4), P3 Neurology (ZapNap, Fall 2025) demonstrated an engagement intensity (0.21\sessions/student/week) exceeding that of some P1 foundational courses (0.15–0.17\sessions/student/week for Biochemistry). Conversely, P3 Psychiatry (MindPath) maintained lower normalized rates (0.02–0.04/sessions/student/week).

Despite lower session counts, qualitative review of PlayLab interaction logs revealed a differential use for P1 and P3 tutors. While P1 students utilized the tutors to stay engaged, have detailed explanations, and request relevant examples, P3 students utilized the tutors for high-level cognitive synthesis. The AI tutors responded effectively to these queries by employing analogies and stepwise explanations to address these conceptual bottleneck topics, consistent with the cognitive load theory (CLT) principles embedded in the tutors’ system design (Table 5). Qualitative query analysis (Table 5) confirmed that P3 students engaged with tutors for targeted, high-level self-assessment and conceptual bottleneck differentiation (e.g., distinguishing delirium from depression) during brief pre-examination windows, rather than extended semester-long tutoring.

2. Academic performance outcomes

**Figure 2:**
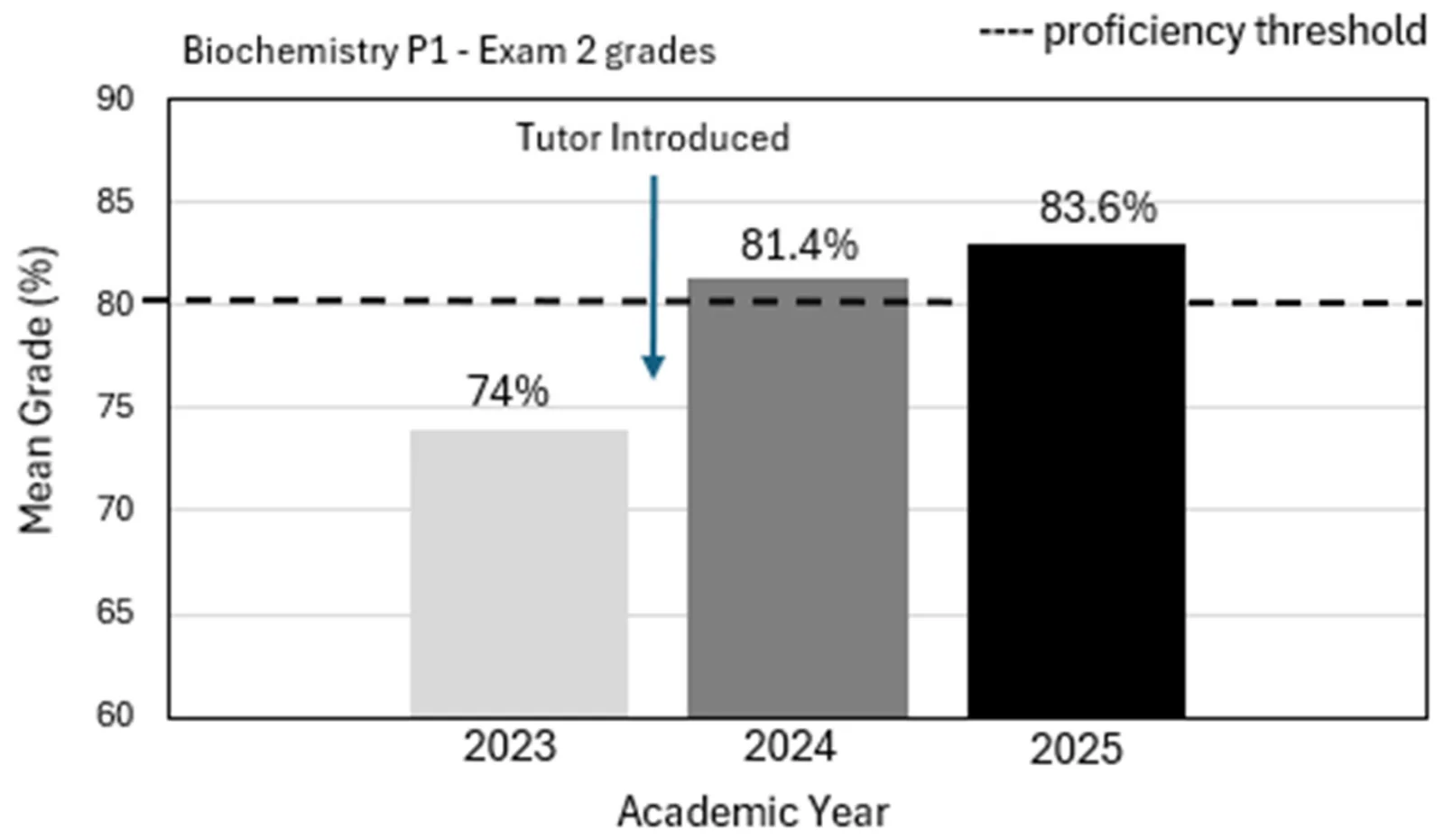
Longitudinal trends in Biochemistry (P1) Exam 2 mean grades by academic year and AI tutor condition (2023–2025). Bar height represents the cohort mean grade (%) on the second biochemistry examination across three academic years, as indicated. Year 2023 is the baseline control cohort, with no AI tutor implemented. The black dashed reference line denotes the institutional 80% benchmark mean grade. Total enrolled cohort: n = 110–119/year.

A. P1 Biochemistry—Longitudinal grade trends: To evaluate the impact of AI tutor integration on summative academic performance, longitudinal grade trends and user versus non-user comparisons were examined across the cohort for the initial pilot deployment of Dr. T’s Tutor (ChatGPT, Fall 2024). Analysis focused on two complementary levels of evidence: (1) a longitudinal comparison of overall Exam 2 mean grades across the three-year observation window (2023–2025) to contextualize the intervention within historical performance trends, and (2) a within-cohort subgroup analysis comparing active tutor users versus non-users in Exam 2 metabolism content to isolate the direct association between tutor engagement and individual academic outcomes. Longitudinal analysis of overall Exam 2 mean grades in the P1 Biochemistry course revealed a consistent upward trajectory following the introduction of AI-assisted tutoring (Figure 2).

B. P1 Biochemistry—User vs. Non-user subgroup analysis: To further examine the relationship between tutor engagement and academic outcomes, a subgroup analysis was conducted within the Fall 2024 cohort (*n* = 32; 16 active users, 16 non-users). Given that Dr. T’s tutor was specifically designed to support metabolism-related content, performance was evaluated using only the examination items directly aligned with tutor-covered material, representing approximately 90% of Exam 2 questions. This targeted scoring approach provides a more precise measure of the tutor’s direct pedagogical impact than overall exam averages, as it isolates the domain in which AI-assisted learning was available to students.

**Figure 3:**
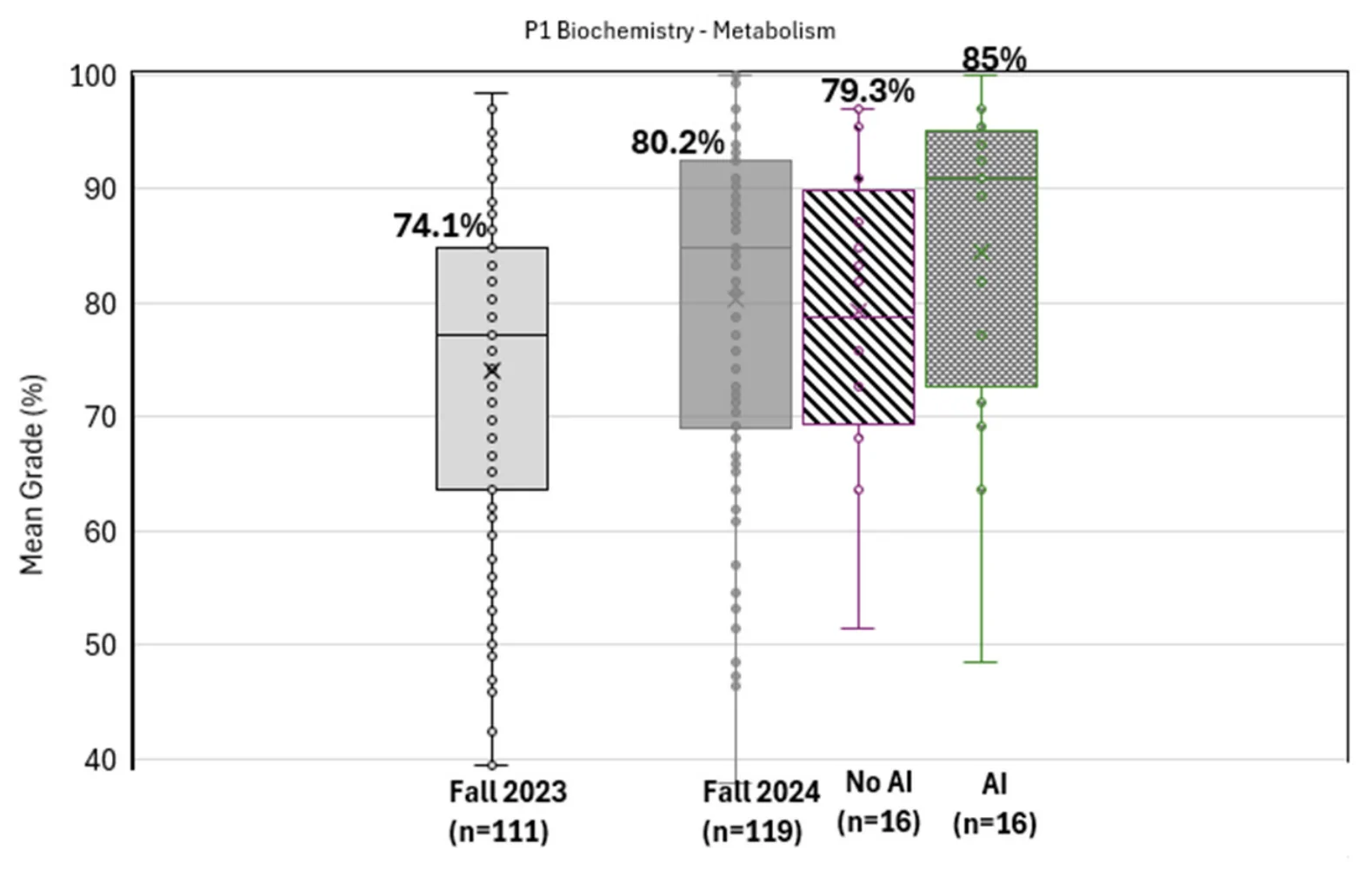
User vs. Non-User Performance Comparison on Metabolism-Aligned Examination Items during Fall 2024 Biochemistry Cohort (*n* = 32). The bar chart displays mean metabolism-related Exam 2 scores across four comparison groups: Fall 2023 baseline cohort (no AI tutor), Fall 2024 cohort (AI tutor), Fall 2024 non-users (Dr. T’s Tutor available but not accessed), and Fall 2024 active users (Dr. T’s Tutor, ChatGPT). Scores reflect metabolism-related assessment items.

Students who actively engaged with Dr. T’s Tutor achieved a mean metabolism-related Exam 2 score of 85%, compared to 79.3% among non-users, a 5.7 percentage point difference that, while considerably higher, did not reach statistical significance, consistent with the pilot sample size (*n* = 32, Figure 3). Notably, a progressive performance gradient was observed across all subgroups. This three-tier gradient is particularly noteworthy, as it suggests that routine, semester-to-semester iterative adjustments in lecture delivery and natural cohort variation contributed to baseline performance gains, with students who did not engage with the tutor in Fall 2024 outperforming the pre-intervention Fall 2023 baseline. Given the quasi-experimental design and concurrent instructional adjustments, longitudinal grade gains cannot be attributed exclusively to AI tutor integration.

**Figure 4:**
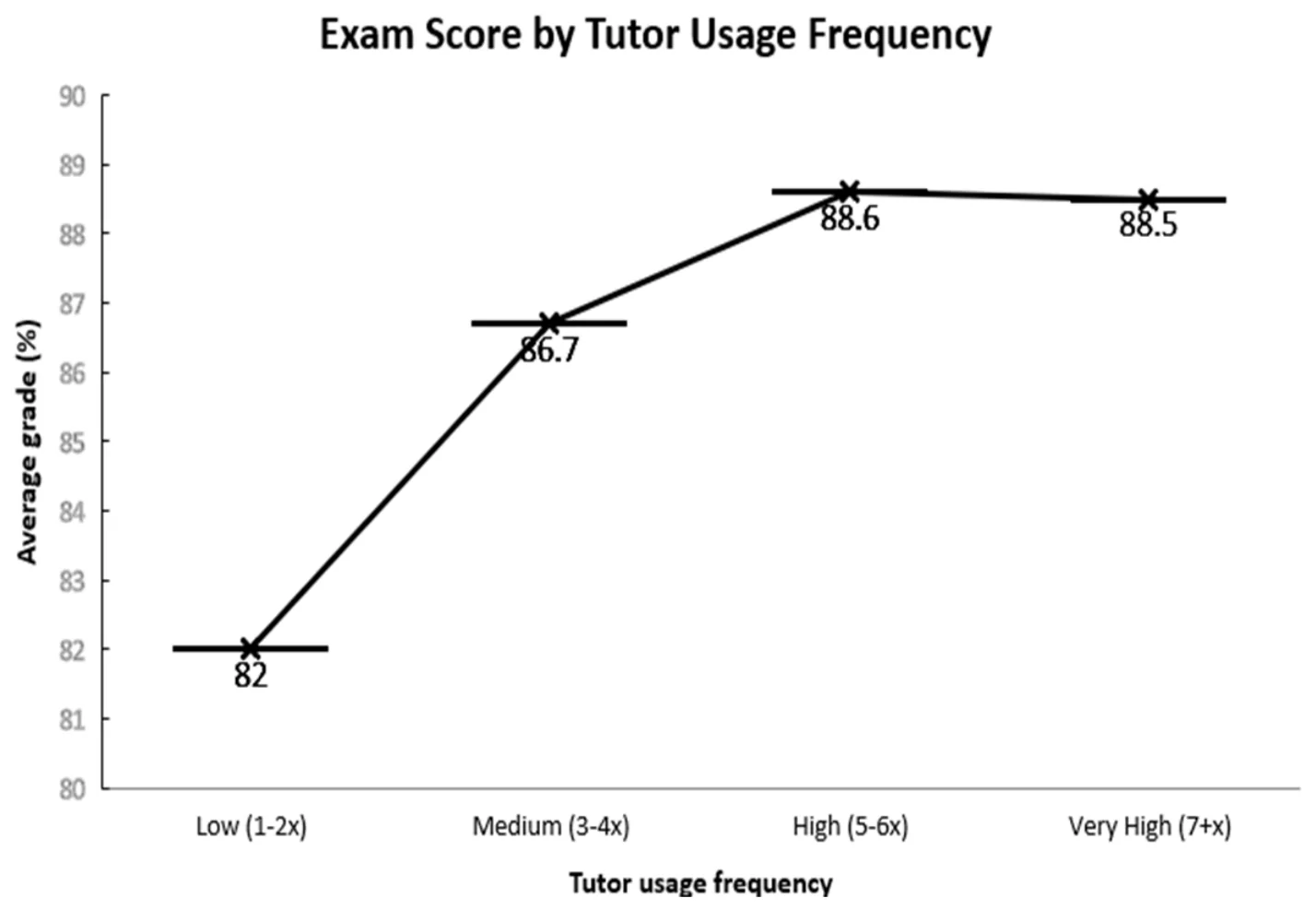
Exploratory examination performance across AI tutor usage frequency tiers. (Fall 2024 P1 Biochemistry Cohort; *n* = 16). The line displays mean metabolism-aligned Exam 2 scores stratified across four self-reported usage tiers: Low (1–2 sessions), Medium (3–4 sessions), High (5–6 sessions), and Very High (7+ sessions) (*n* = 3–5/group).

Furthermore, examination scores exhibited a tiered distribution when grouped by platform engagement levels (Figure 4). Students in the low-usage tier (1–2 sessions) achieved a mean score of 82.0%, while those in the medium (3–4 sessions), high (5–6 sessions), and very high (7+ sessions) tiers achieved means of 86.7%, 88.6%, and 88.5%, respectively. While these differences did not reach statistical significance, given the pilot sample size, the consistent directional trend across all usage tiers is noteworthy.

**Figure 5:**
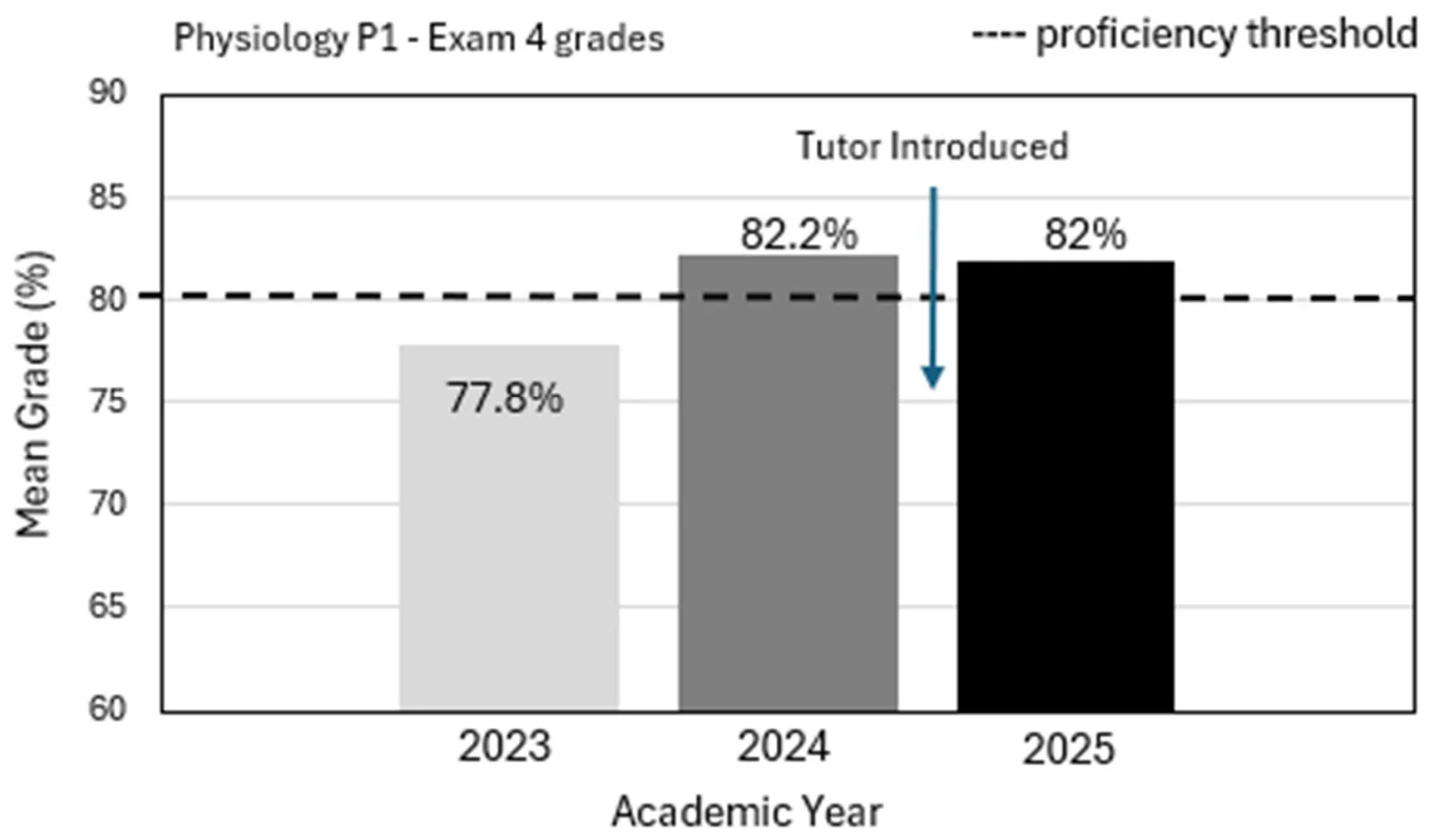
Longitudinal trends in Physiology (P1) Exam 4 mean grades by academic year and AI tutor condition (2023–2025). Bar height represents the cohort mean grade (%) on the fourth physiology examination across three academic years, as indicated. Year 2023 is the baseline control cohort, with no AI tutor implemented. The black dashed reference line denotes the institutional 80% benchmark mean grade. Total enrolled cohort: n = 111–119/year.

C. P1 Physiology—In the P1 Physiology course, the pre-intervention cohort (Fall 2023) recorded a mean Exam 4 score of 77.8%. Following the introduction of ANSwer Bot (PlayLab, Fall 2025), the cohort mean increased to 82%, representing a 4.2 percentage point improvement (Figure 5).

D. P3 Clinical Modules—Neurology and Psychiatry: In the P3 Neurology course, mean course grades remained stable across the three-year observation window: 88.2% (2023, no AI), 86.3% (2024, no AI), 86.1% (2025, AI introduced). While no grade improvement was observed in the intervention year, it is important to note that engagement was limited to 90 sessions within a compressed four-week module, and all cohort means remained well above the 80% proficiency threshold. Similarly, in the P3 Psychiatry course, mean grades were 84.3% (2024), 83.7% (2025, MindPath introduced), and 85.1% (2026, with AI).

Across both P3 courses, all cohorts maintained performance above the 80% benchmark, suggesting that the AI tutors may have contributed to performance stabilization in high-achieving clinical cohorts where ceiling effects limit the magnitude of measurable grade improvement.

E. AACP Proficiency Outcomes and Trends—AACP standardized proficiency data were obtained for all the courses involved from Fall 2023 to Spring 2026. In the P1 Biochemistry course (pilot study), longitudinal analysis for the standardized proficiencies across relevant domains (4300-05, 06, 08) revealed a meaningful upward shift in overall course performance following AI tutor integration (Table 6). The pre-intervention cohort (Fall 2023) recorded a course-wide mean proficiency below the standardized 80% proficiency target. This rose in Fall 2024 (ChatGPT) and was sustained in Fall 2025 (PlayLab), with both intervention cohorts exceeding the 80% benchmark.

Targeted analysis of the three Biochemistry proficiency domains directly aligned with Dr. T’s Tutor’s curated knowledge base revealed differential gains across domains. The three tutor-aligned domains demonstrated positive longitudinal trends from Fall 2023 to Fall 2025.

Longitudinal analysis of standardized proficiency across relevant domains in the P1 Physiology course revealed no major changes in overall course performance following AI tutor integration in Fall 2025 (Table 6).

Longitudinal analysis of standardized proficiency across relevant domains in the P3 Neurology module revealed a substantial increase in overall course performance following AI tutor integration in Fall 2025 for both proficiencies (Table 6).

Finally, the longitudinal analysis of standardized proficiency across relevant domains in the P3 Psychiatry module revealed an increase in overall course performance for both the pathophysiology (5368-01) and pharmacology content (53680-03) following AI tutor integration in Spring 2025 (Table 6).

3. Student perception, satisfaction, and qualitative findings.

To complement the quantitative performance outcomes, student perceptions of the AI tutors were evaluated through a mixed-methods approach encompassing Likert-scale satisfaction ratings, Pearson correlation analyses, and inductive thematic analysis of open-ended survey responses. Together, these data provide insight into not only whether the AI tutor was associated with improved academic outcomes, but also how students experienced and engaged with it as a learning tool.

**Figure 6:**
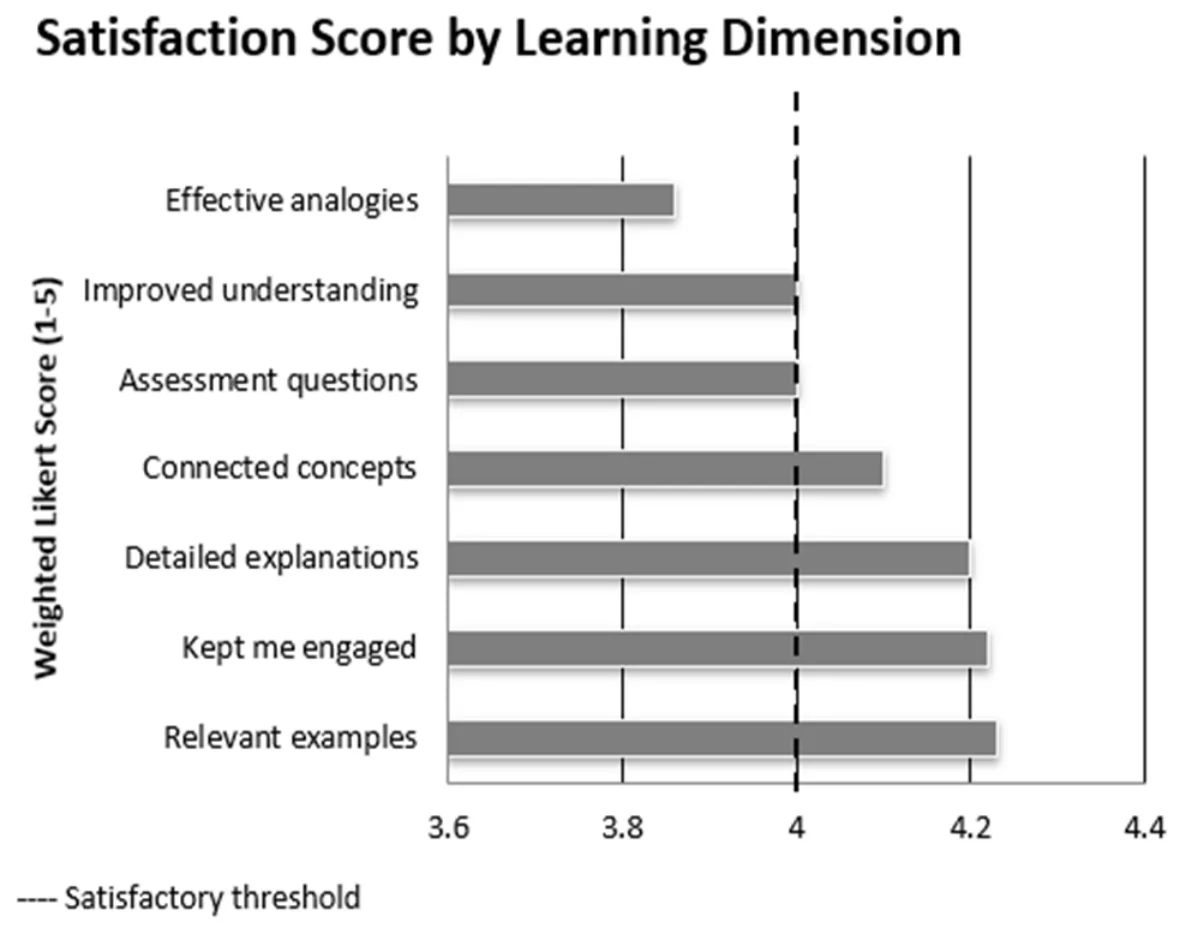
Student Satisfaction Ratings by Pedagogical Learning Dimension. Horizontal bars display mean weighted Likert scores (1–5 scale) across seven pedagogical dimensions evaluated by active tutor users. The vertical dashed reference line at 4.0 denotes the satisfactory threshold between “Good” and “Strong” performance tiers.

A. Quantitative satisfaction ratings—Student satisfaction with Dr. T’s Tutor was assessed across several learning dimensions using a five-point Likert scale (Fall 2024). Ninety percent of respondents agreed or strongly agreed that the platform was easy to use, and 83% found the tutor’s responses clear and understandable. Accessibility was rated positively by 82% of students, reflecting the 24/7 availability of the tool. Six out of seven dimensions exceeded the satisfactory threshold of 4.0/5.0, reflecting broadly positive student perceptions of the tutor’s pedagogical utility (Figure 6).

Pedagogically, the highest-rated dimensions were “Relevant Examples” (4.23/5.0) and “Kept Me Engaged” (4.22/5.0), suggesting that students particularly valued the tutor’s ability to contextualize abstract content through applied examples and to sustain motivation during independent study. “Effective Use of Analogies” (3.86/5.0) was the only dimension rated below 4.0 threshold, identifying a specific area for iterative improvement in future bot design iterations.

B. Satisfaction and Usage Correlation analysis—To examine whether students who found Dr. T’s Tutor more useful or used it more frequently also performed better academically, Pearson correlation analyses were conducted among composite satisfaction scores, usage frequency, and Exam 2 performance (*n* = 16 active users). Positive directional trends were observed for both relationships: composite satisfaction and exam scores (r = 0.19, *p* = 0.489) and usage frequency and exam scores (r = 0.21, *p* = 0.442). Neither correlation reached statistical significance; however, the consistent positive directionality across both metrics suggests a meaningful trend that warrants investigation in larger, adequately powered cohorts.

C. Qualitative thematic analysis—Students primarily used AI tutors for on-demand clarification, integration of concepts across lectures, self-testing, and reorganization of complex content. Students emphasized the value of receiving rapid explanations during independent study, particularly in the evenings and on weekends, and several described the tutor as helpful for addressing “small questions” that did not require direct professor input. Students also reported using the tool to connect material across lectures, generate practice questions, and simplify difficult mechanisms and pathways.

The most frequently cited theme was overall satisfaction and positive experience, with students describing the tools as “*amazing*,” “*incredibly useful*,” and “*easily accessible*.” Thematic analysis of open-ended survey responses to why students used the tutor and the standard course evaluation comments (*n* = 46 total comments) revealed several main recurring themes across all AI tutor deployments, analyzed inductively using a framework approach.

Conceptual understanding and clarification emerged as the primary academic driver of use, with students reporting that the tutor helped them “*connect the different lectures together rather than sifting through the hundred or so slides given*” and provided “*simple explanations and appropriate examples*” for difficult topics. Accessibility and convenience were the second most common motivator, with students specifically citing late-night and weekend availability as critical to their independent study workflow, a finding consistent with the on-demand, asynchronous design of the tutors (Table 7).

Self-testing and knowledge assessment reflected students’ use of the tutor to generate practice questions and evaluate their own understanding prior to examinations. As for limitations and suggestions for improvement, those were centered primarily on ChatGPT-specific barriers, including account creation requirements, daily message limits, file upload restrictions, perceived subscription costs, and concerns about AI accuracy. These findings directly justified the subsequent transition to PlayLab as the primary deployment platform, which eliminated subscription barriers and provided instructor access to anonymized interaction logs for ongoing evaluation.

Interaction log analysis for PlayLab-based deployments corroborated these qualitative themes for all AI tutors, revealing that the most common student query types involved stepwise explanations, concept integration across lecture modules, and generation of self-assessment questions, patterns consistent with the “on-demand clarification” and “self-testing” themes identified in the survey data.

## 4. Discussion

The primary challenge related to student tutoring identified by Bloom [1] was the impossibility of providing individualized one-on-one tutoring to every student in a large-enrollment course. Our implementation of four distinct AI tutors, Dr. T’s Tutor, ANSwer Bot, ZapNap, and MindPath, demonstrates that generative AI can approximate the core mechanisms of effective tutoring, thus providing personalization, immediate feedback, and 24/7 availability.

AI-assisted tutoring can approximate the individualized instruction effect described by Bloom’s 2 Sigma Problem [28], even within large pharmacy cohorts where one-on-one human tutoring is logistically infeasible. Herein, we reported that active users of Dr. T’s Tutor achieved a higher score in the assessment compared to non-users. While not reaching statistical significance, this finding is directionally consistent with the performance advantages associated with individualized instruction. Furthermore, a stratified usage pattern was observed across usage tiers, with students in the highest engagement tier (7+ sessions) achieving higher mean scores compared to low-frequency users (1–2 sessions).

Previous studies [1] demonstrated that students receiving one-on-one human tutoring outperformed conventionally instructed peers by approximately two standard deviations. While our pilot data cannot claim equivalence to this effect size, the observed performance gradient across usage tiers is consistent with the mastery learning principle, which states that increasing time-on-task with individualized feedback produces cumulative academic gains. The non-significant correlation obtained precludes definitive conclusions regarding a true underlying effect. The small pilot sample limits statistical power, and the observed directional trend should be interpreted cautiously as hypothesis-generating rather than confirmatory, underscoring the need for a larger, adequately powered replication study.

Sustained AI tutor integration is associated with meaningful longitudinal improvements in both summative exam performance and standardized proficiency outcomes, with the greatest gains observed in domains directly aligned with the tutor’s curated knowledge base. Regarding summative outcomes, exam 2 mean grades in the P1 Biochemistry course rose steadily from Fall 2023 (no AI) to Fall 2024 (ChatGPT) and Fall 2025 (PlayLab), with a cumulative improvement of 9.6 percentage points over the observation window.

The improvement observed in P1 Physiology following the introduction of ANSwer Bot is consistent with the pattern identified in Biochemistry and further supports the hypothesis that AI-powered personal tutors can meaningfully enhance student performance in foundational science courses. The pre-intervention cohort (Fall 2023) recorded a mean Exam 4 score of 77.8%, falling below the institutional 80% proficiency benchmark. Following the deployment of ANSwer Bot (PlayLab, Fall 2025), the cohort mean rose to 82.0%, representing a 4.2 percentage point improvement and crossing the benchmark threshold. This trajectory mirrors the 2 Sigma framework proposed by Bloom [1], wherein individualized instructional support, even when delivered at scale through AI, can shift student performance distributions toward mastery-level outcomes. The fact that this improvement occurred in a course with relatively modest engagement (90 sessions) suggests that even limited, targeted AI tutor use may be sufficient to produce meaningful gains in foundational content, particularly when the tutor is tightly aligned to course-specific learning objectives.

In the P3 Neurology course, mean course grades remained stable across the three-year observation window: 88.2% (2023, no AI), 86.3% (2024, no AI), and 86.1% (2025, ZapNap introduced), with no statistically meaningful grade improvement observed in the intervention year. These findings warrant careful contextual interpretation. First, engagement with ZapNap was limited to 90 sessions within a compressed four-week clinical module, a structural constraint that substantially reduced the opportunity for AI-mediated learning to influence summative performance. Second, and perhaps more importantly, the pre-existing cohort means in Neurology were already well above the 80% institutional benchmark, suggesting a ceiling effect that limits the magnitude of detectable grade improvement regardless of instructional intervention. This phenomenon is consistent with the broader educational literature on high-achieving cohorts, wherein performance gains are inherently constrained by the upper bounds of assessment design [29]. Taken together, the Neurology data should not be interpreted as evidence of inefficacy; rather, they reflect the challenge of demonstrating additive value in cohorts that have already achieved mastery-level performance. Future studies should consider alternative outcome measures, such as time-to-mastery, self-efficacy, or performance on higher-order clinical reasoning items, that may be more sensitive to AI tutor effects in advanced clinical populations.

A similar pattern of performance stabilization was observed in the P3 Psychiatry course. Mean course grades were 84.3% (2024, no AI), 83.7% (2025, MindPath introduced), and 85.1% (2026, with continued AI access), reflecting a modest but positive trajectory following the initial year of implementation. The slight decline observed in the first year of AI introduction (2025) is consistent with early adoption dynamics documented in educational technology research, wherein students and faculty require an acclimation period before the full benefits of a new instructional tool are realized [30]. The subsequent recovery and improvement in 2026, representing a 1.4 percentage point gain over the pre-intervention baseline, suggests that sustained access to MindPath may support incremental performance gains over time, even within the constraints of a compressed clinical module. As with Neurology, all Psychiatry cohort means remained above the 80% proficiency threshold throughout the observation period, reinforcing the interpretation that AI tutors in P3 clinical settings may function primarily as performance stabilizers and confidence-building tools rather than as drivers of large-scale grade improvement. This distinction has important implications for how the success of AI tutoring interventions is defined and measured in advanced clinical pharmacy education.

Thus, across all four courses examined (Biochemistry, Physiology, Neurology, and Psychiatry), a coherent pattern emerges that reflects both the promise and the contextual complexity of AI-powered personal tutoring in pharmacy education. In P1 foundational science courses, where students are acquiring core biochemical and physiological knowledge and where pre-intervention performance was below the 80% benchmark, AI tutors were associated with meaningful, measurable grade improvements. In P3 clinical courses, where cohort means were already above benchmark and module duration was compressed, AI tutors appear to have contributed to performance stabilization rather than dramatic improvement. This distinction is not a limitation of the intervention but rather a reflection of the differential needs of learners at different stages of professional formation. These findings collectively suggest that the impact of AI-powered personal tutors is moderated by baseline performance level, course structure, and engagement opportunity, and that outcome metrics should be selected accordingly when evaluating AI tutoring efficacy across diverse curricular contexts.

The differential engagement in some of the foundational courses is consistent with the structure of P1 courses, though the pattern differed meaningfully between the two subjects. In Biochemistry, the semester-long course structure features multiple comprehensive examinations in which foundational metabolic content is revisited and reinforced across successive assessment points, providing repeated incentives for students to seek supplemental support throughout the term. By contrast, the Physiology content covered by ANSwer Bot was assessed exclusively on the course’s fourth and last examination, concentrating the window of relevant study into a single, high-stakes event. The 90 sessions recorded for ANSwer Bot (Physiology, 2025), therefore, represent strong engagement given this structural constraint, as students accessed the tutor in a more targeted, pre-exam fashion rather than as a sustained study companion across the semester.

As for the structure of P3 modules, they are fast-paced four-week modules in which the content covered by each AI tutor is assessed only once, providing a narrower window for repeated engagement compared to semester-long P1 courses. Taken together, these engagement patterns suggest that the AI tutors served distinct but complementary functions across curricular levels: as a sustained retrieval and reinforcement tool in P1, and as a targeted, on-demand high-level resource in P3.

A critical factor moderating student engagement in the advanced P3 modules (ZapNap and MindPath) was the intentional implementation of a strict “clinical fence”. System prompts were programmed to restrict responses strictly to basic pathophysiology and pharmacology, explicitly prohibiting individualized diagnostic, dosing, or therapeutic recommendations and redirecting students to clinical faculty for patient case discussions. While this design safeguard was essential to prevent automation bias, avoid clinical hallucinations, and ensure patient safety boundaries, it might have introduced a recognized pedagogical tension regarding perceived usefulness. Third-year pharmacy students primarily seek academic support when synthesizing complex, patient-specific pharmacotherapeutic regimens and evaluating clinical case scenarios. Restricting the bots from assisting with clinical decision-making likely diminished their perceived value relative to primary literature, clinical guidelines, and faculty office hours.

Furthermore, this boundary reflected necessary institutional and interprofessional governance dynamics. Interdisciplinary clinical faculty were initially hesitant to participate in developing a comprehensive AI tutor integrating both medicinal chemistry and clinical therapeutics, expressing discomfort with AI capabilities and requesting strict system boundaries to ensure the tools would not infringe upon clinical decision-making content. This faculty reluctance mirrors national pharmacy education data indicating that widespread AI adoption is constrained by faculty unfamiliarity and concerns over assessment integrity. Thus, the “clinical fence” served a dual purpose: it maintained essential ethical and patient safety guardrails while establishing the institutional trust necessary for preliminary AI deployment. Future iterations should explore faculty-guided, case-based simulation frameworks where AI provides structured Socratic feedback on student-generated clinical rationale without independently offering treatment recommendations.

Concurrently with assessment grades, we analyzed AACP proficiency domains related to the content on those created bots. Mean performance across the four tutor-aligned AACP proficiency domains also rose from the control to the intervention cohorts, approaching the standardized 80% proficiency benchmark. The progressive nature of these gains, with each successive cohort outperforming the last, is consistent with the “implementation dip and recovery” pattern described in educational technology adoption literature [31], wherein early-adopter cohorts experience modest gains as both students and instructors learn to use the tool effectively, while subsequent cohorts benefit from a more refined and trusted intervention. The fact that these gains are reflected not only in internal examination scores but also in AACP-reported proficiency data confers institutional validity that extends beyond a single classroom study, positioning these findings as directly relevant to pharmacy program accreditation and benchmarking efforts at the national level. Importantly, however, the use of exam-specific outcome measures, rather than course-wide AACP proficiency aggregates, represents a deliberately conservative and construct-valid approach to isolating the direct impact of AI tutor engagement on student performance. Because AACP proficiency scores reflect the aggregated performance across all examination items tagged to a given domain throughout the entire course, including assessments on which the AI tutor’s content was not directly tested, they capture a broader curricular footprint than the targeted exam snapshots used as primary outcome measures in this study. The convergence of both metrics in the same directional trend therefore strengthens, rather than complicates, the overall interpretation of these findings. It is worth noting that for all the AACP proficiency data, the number of assessments included in the specific proficiencies analyzed, as well as the number of items per assessment, was equivalent for each cohort in all courses and modules.

A significant portion of the knowledge base for our tutors consisted of recorded lecture videos and transcripts. According to AACP guidelines, educational videos are most effective when embedded within a context of active learning [32]. Our AI tutors transformed the traditionally passive experience of watching a recorded lecture into an active, conversational one. Grounding AI tutor knowledge bases in instructor-recorded lecture content transforms passive video consumption into active, inquiry-driven learning, directly addressing AACP principles of guided self-evaluation.

In our study, qualitative log analysis revealed that students frequently queried the tutors with content-specific questions referencing lecture material, such as clarifying a specific pathway explained at a particular point in a lecture. This pattern was corroborated by the thematic analysis, in which “conceptual understanding and clarification” and “self-testing and knowledge assessment” emerged as the primary academic drivers of tutor use. Previous research [32,33,34] already identified guiding questions and active processing as the critical mechanisms by which video-based instruction achieves learning gains equivalent to live instruction. By allowing students to cross-examine lecture content in real time, asking the AI to clarify, expand, or connect concepts from specific videos, the tutor functioned as a cognitive navigator for the media, ensuring that passive viewing was converted into active sense-making. This directly addresses the AACP principle of using guiding questions to support self-evaluation of understanding [32], while also providing immediate corrective feedback that prevents misconception consolidation.

The transition from a ChatGPT deployment (Fall 2024) to a PlayLab-based specialized tutor (Fall 2025) was associated with an increase in adoption, a further improvement in grades, and a rise in the proportion of students reporting improved biochemistry understanding. Critically, the decision to disable web search within the PlayLab tutors was a deliberate pedagogical choice to reduce hallucination and ensure that student queries were answered exclusively within the framework of instructor-verified course content. The “satisficing” design philosophy underlying our specialized bots, constraining the AI to course-specific transcripts and videos rather than the open web, directly addresses the hallucination risk that represents the most frequently cited barrier to AI adoption in medical education [35,36]. By ensuring that the AI functions as a mirror of instructor expertise rather than a generic information retrieval system, we provide a model for responsible AI integration that is replicable across pharmacy and health sciences curricula.

LLMs have been shown to have high potential in pharmacy and medical education contexts [37], with a need for careful implementation, content constraints, and pedagogical boundaries. Looking forward, the modular nature of the PlayLab platform creates the possibility of bot-stacking across foundational science courses, enabling a unified AI tutoring ecosystem for P1 students spanning Biochemistry, Physiology, and beyond within a single interface.

Another point in this study is related to equity and accessibility of these instruments in Pharmacy Education. Platform selection is not merely a technical decision but an equity imperative. AI tutoring tools that require paid subscriptions or complex account creation create differential access that undermines the universal benefit potential of the intervention. Our thematic analysis identified “non-use and barriers to access” as well as “limitations and suggestions for improvement”. Those were recurring themes, with students specifically citing ChatGPT account creation requirements, daily message limits, and perceived subscription costs as deterrents to adoption. These findings directly pointed to the decision to transition to PlayLab, a subscription-free, education-focused platform for all subsequent deployments, a platform change that was driven entirely by student feedback rather than faculty preference.

If AI tutoring is to approximate the 2 Sigma effect at scale, it must be universally accessible regardless of students’ socioeconomic status. The equity rationale for platform migration is supported by a growing body of literature on the “digital divide” in higher education, which demonstrates that technology-mediated interventions disproportionately benefit students who already have access to premium tools, thereby widening rather than closing achievement gaps when access is unequal [38,39]. In professional education pipelines such as pharmacy, where workforce diversity has direct implications for health equity, ensuring universal access to educational innovations is not merely a pedagogical concern but a social justice imperative [40].

This study also points to changing roles for educators. AI tutor integration redefines the faculty role by offloading routine content delivery to the AI, thereby freeing instructors to focus on the high-order mentoring, professional socialization, and ethical deliberation that only human educators can provide. The qualitative finding that students primarily used Dr. T’s Tutor for “small questions” during evenings and weekends, interactions that would otherwise require asynchronous email exchanges or remain unresolved until the next class session, suggests that the AI effectively absorbed a category of low-complexity, high-frequency faculty interactions. With over 300 sessions recorded just in the P1 Biochemistry cohort alone, the volume of student–AI interactions represents a substantial reallocation of cognitive labor from instructor to tool. This reallocation is consistent with the “cognitive offloading” principle in educational psychology [41], which holds that when routine procedural tasks are delegated to external tools, human cognitive resources are freed for higher-order processing. In the context of pharmacy education, this means that faculty time previously spent re-explaining the Krebs cycle as an example can be redirected toward clinical reasoning, professional identity formation, and the mentoring relationships that research consistently identifies as the most transformative human elements of health professions education.

One important characteristic of our bots is the need to navigate foundational and clinical boundaries. A “foundational-first” scoping strategy, deliberately limiting AI tutor content to pathophysiology and pharmacological mechanisms while excluding clinical decision-making, mitigates the risk of inappropriate AI reliance and provides a replicable blueprint for responsible AI integration across health professions curricula. The explicit redirection protocol embedded in all four tutors, exemplified by responses such as “*I can explain the mechanism of SSRIs, but for clinical dosing in a specific patient case, please consult your clinical instructor*,” established a clear and consistent boundary between AI-supported foundational learning and human-led clinical synthesis. This boundary was reinforced by the decision to ground all tutor knowledge bases exclusively in instructor-verified course content, preventing students from using the AI as a substitute for clinical judgment. A common critique of AI in medical education is the risk of students outsourcing clinical reasoning to algorithmic systems, a concern that is particularly acute in pharmacy education, where medication errors carry direct patient safety implications. Our foundational-first approach directly addresses this concern by positioning the AI as a mastery learning scaffold for the “building blocks” of pharmaceutical science, while explicitly preserving high-stakes clinical synthesis for human-led instruction. This scaffolded model is consistent with Vygotsky’s Zone of Proximal Development [42], in which the AI functions as a “more knowledgeable other” for foundational content while human instructors retain authority over the complex, context-dependent reasoning that defines clinical practice. AI’s highest value in healthcare lies not in replacing human judgment but in augmenting it [43], freeing professionals to focus on the empathetic, relationship-centered dimensions of care that only humans can provide. This principle extends naturally to pharmacy education, where AI should support foundational learning while clinical decision-making remains firmly within the domain of human expertise [44].

### 4.1. Limitations

While the findings of this pilot study are promising, several limitations warrant acknowledgment. The non-significant correlation between usage frequency and exam performance reflects the exploratory nature of this pilot analysis. Given the small sample size, statistical power was insufficient to detect small-to-moderate effects; accordingly, the observed directional trends should be interpreted as preliminary and hypothesis-generating, pending confirmation in a larger, adequately powered study.

The adoption rates are mostly survey response-derived and likely underestimate true platform utilization, as voluntary survey participation introduces selection bias; future iterations will leverage PlayLab’s administrative dashboard to report log-verified adoption rates independent of survey completion. Additionally, the compressed four-week structure of P3 modules limits the comparability of P1 and P3 engagement metrics; future analyses should employ time-normalized engagement measures to enable equitable cross-cohort comparisons.

Several other methodological considerations warrant cautious interpretation of the academic performance gains described. First, because participation was voluntary, self-selection bias represents a potential confounder. Students who actively engage with supplemental AI tutors may possess higher baseline academic motivation, superior self-regulation, or different study habits compared to non-users. While active users outperformed non-users on metabolism-aligned assessments in the pilot cohort, these preliminary data reflect a positive association rather than definitive causality. Second, although item-level difficulty analyses confirmed examination rigor was comparable across the 2023–2025 cohorts and primary instructional faculty remained constant, subtle iterative refinements in lecture delivery might have occurred and coincided with AI deployment. Future multi-center investigations utilizing matched-cohort designs or baseline academic covariates (e.g., prior GPA or pre-assessment benchmarking) will be essential to fully isolate AI-specific learning gains from baseline student motivation.

Another methodological consideration inherent to multi-year generative AI implementations is the rapid evolution of underlying foundation models and software interfaces. Although all four tutors were strictly constrained to static, instructor-verified course materials via RAG to prevent out-of-scope drift, the underlying foundation models and hosting architecture evolved between Fall 2024 and Spring 2026. Upgrades in base model reasoning, context processing fidelity, and conversational scaffolding, alongside the operational transition from individual ChatGPT accounts to the centralized PlayLab platform, may have subtly improved response quality, reduced user friction, and influenced adoption rates over time. While course syllabi, core learning objectives, and examination structures remained standardized, this shifting technological baseline represents a concurrent co-intervention that should be accounted for in longitudinal AI educational research.

Looking forward, the AACP proficiency data, already reported to a national accrediting body, presents a unique opportunity for multi-institutional replication. Inviting other pharmacy programs using comparable AI tutoring tools to contribute their proficiency data to a shared analysis would transform this pilot into a landmark multi-site investigation with the statistical power to detect meaningful effect sizes and establish national benchmarks for AI-enhanced pharmacy education.

The observed exploratory engagement gradient relationship, where increased tutor engagement correlated with progressively higher examination performance across all four usage tiers, is directionally consistent with mastery learning principles and the individualized instruction effects described by Bloom [1]. However, the pilot sample size (*n* = 16) precludes a definitive causal interpretation, as this pattern may partially reflect self-selection bias. These preliminary findings therefore suggest a positive association warranting investigation in larger, randomized cohorts with sufficient statistical power to disentangle engagement effects from pre-existing academic motivation.

Regarding the P3 cohorts, the absence of measurable grade improvement following ZapNap and MindPath introduction is most prudently explained by a ceiling effect, as pre-intervention cohort means were already high (≥80%). Importantly, item-level difficulty analysis confirmed that examination rigor was comparable across the 2023–2025 observation window, ruling out the alternative explanation that the 2025 cohort simply encountered a more challenging assessment. Taken together, these findings suggest that AI-mediated performance gains may be most pronounced in foundational courses where students historically perform below the 80% proficiency benchmark, and that in advanced clinical modules where performance is already optimized, AI tutors may serve a maintenance rather than an elevating function, a distinction with meaningful implications for strategic tutor deployment across pharmacy curricula.

### 4.2. Future Directions

Future iterations of this work should focus on the transition from text-centric tutoring to a multimodal AI ecosystem that more fully addresses the 2 Sigma Problem through the lens of Universal Design for Learning (UDL, [45]). While the current RAG-based tutors provide high-quality text interactions, the integration of other tools like Notebook for AI-generated study podcasts and PlayLab for interactive, scaffolded apps will allow for “multiple means of representation”, a core pillar of UDL. By providing content in varied formats, such as audio summaries for students to utilize during commutes or interactive “sandbox” simulations for kinesthetic learners, we can more closely replicate the adaptive, flexible nature of a 1:1 human tutor who adjusts their delivery based on a student’s immediate needs. In the demanding context of pharmacy education, these multimodal tools offer the necessary flexibility to support diverse learning styles and reduce cognitive load [41], ensuring that personalized mastery learning is not only scalable but also truly equitable and inclusive. Ultimately, this multimodal approach represents the next step in “humanizing” digital education, providing a digital mentor that meets every student where they are, regardless of their preferred learning modality.

Future investigations should also prioritize the expansion of this AI tutoring framework to a multi-institutional scale. The AACP proficiency reporting system, a standardized, nationally collected outcome measure already embedded in pharmacy curricula, offers a uniquely accessible and low-burden framework for cross-institutional comparison. Pharmacy faculty currently utilizing PlayLab or ChatGPT-based tutors, regardless of prior research experience, could contribute proficiency outcome data to a shared analysis, enabling a statistically powered examination of the 2 Sigma effect across diverse institutional contexts, student populations, and therapeutic content areas. Given that the present pilot demonstrated mean proficiency gains in tutor-aligned domains over three cohorts, a multi-site study would provide the statistical power necessary to detect meaningful effect sizes and establish national benchmarks for AI-enhanced pharmacy education.

The progressive improvement in proficiency outcomes observed across the three-year observation window, with overall cohort means rising and approaching the standardized 80% benchmark across tutor-aligned domains, demonstrates the feasibility of scaling this framework across the full P1–P4 curriculum. Future research could apply this standardized proficiency framework to additional courses while addressing the engagement variance observed between foundational and clinical years. As shown in the engagement data, P1 cohorts generated substantially higher interaction volumes compared to P3 clinical cohorts, suggesting that future implementations should investigate the structural and motivational factors that drive differential engagement across training levels and develop targeted strategies to increase AI tutor utilization.

Central to this scaling effort will be the systematic analysis of PlayLab interaction logs to iteratively refine the “clinical fence” for higher-level rotations, including P4 Advanced Pharmacy Practice Experiences (APPEs). By identifying recurring student attempts to seek diagnostic or treatment advice, instructors can tune system prompts to provide more nuanced clinical reasoning support while maintaining strict pedagogical boundaries, a process consistent with best practices in clinical AI implementation [44]. This iterative refinement process will also generate a rich qualitative dataset on the types of clinical questions pharmacy students ask during self-directed study, providing valuable insights for curriculum development and faculty professional development.

Ultimately, these efforts aim to transition from isolated pilot interventions toward a program-wide AI literacy competency framework. Such a framework would align with emerging AACP standards, moving beyond simple content recall to evaluate students’ ability to responsibly and critically engage with AI as a collaborative tool in pharmacy practice. Additionally, by developing these tools and allowing students to interact with AI early in their professional formation, we support the development of AI competencies that will be directly applicable to their future careers, a recognized priority by AACP, which emphasizes that student pharmacists “are going to be pharmacists who utilize these AI tools in the healthcare systems, and that’s a skill set they need to develop” [22]. Realizing this vision will require sustained faculty buy-in, institutional support for iterative bot development, and a collaborative culture in which basic science and clinical faculty co-design AI tools that scaffold foundational knowledge while preserving the irreplaceable human dimensions of pharmacy education [43].

## 5. Conclusions

The “2 Sigma Problem” has challenged educators for four decades: how do we deliver the individualized, mastery-oriented instruction of a personal tutor to every student in a large-enrollment course? This study demonstrates that generative AI, when thoughtfully designed and deliberately constrained to instructor-verified content, offers a scalable and equitable approximation of that ideal. By deploying four course-specific AI tutors across P1 and P3 pharmacy cohorts at the University of Houston College of Pharmacy, we provide preliminary evidence that curriculum-aligned AI tutoring can meaningfully support student learning without compromising pedagogical safety or institutional integrity.

The data presented here are encouraging. Grades rose from the pre-intervention to AI tutor deployment cohorts, while overall AACP proficiency means increased, surpassing the standardized 80% benchmark. Across all cohorts and courses, student satisfaction remained high, with 100%, in certain course respondents agreeing that the tutor improved their understanding of course material.

These findings, while preliminary, establish a replicable framework for responsible AI integration in pharmacy education, one grounded in cognitive load theory, RAG-based curricular fidelity, and a deliberate “foundational-first” approach that preserves the irreplaceable human dimensions of professional formation. As AI tools become standard in healthcare delivery, pharmacy programs have both an opportunity and an obligation to prepare students not merely to use these technologies, but to engage with them critically, responsibly, and with clinical judgment intact [22,23,24]. The tutors described here represent a first step toward that vision, and an invitation to the broader pharmacy education community to build upon it.

## Figures and Tables

**Table 1:** Summary of Included Pharmacy Courses.

Course Code & Semester	Course Title	Credits	Prerequisites & Co-Requisites	Level	Short Description Target Outcome Measure
**PHAR 4300** **Fall**	Biochemistry	3	First-year standing	P1	Semester-long. Explores the biochemical basis of health and disease, focusing on metabolism. Target: Exam 2
**PHAR 4320** **Fall**	Physiology I	3	First-year standing	P1	Semester-long. Comprehensive study of the central/autonomic nervous system, endocrine, and skin physiology as a foundation for clinical assessment. Target: Exam 4
**PHAR 5335** **Fall**	Integrated Neurology Module	3	Third-year standing	P3	Modular, 4-week long. Integrated clinical management of neurological conditions across pathophysiology and therapeutics. Target: Course average
**PHAR 5368** **Spring**	Integrated Psychiatric Module	3	Third-year standing	P3	Modular, 4-week long. Integrated clinical management of psychiatric diseases across pathophysiology and therapeutics. Target: Course average

**Table 2:** AI Tutor deployments by course, student level, and cohort.

Student Level	Bot Name (Platform)	Course/Content Area	Academic Year	Enrollment (*n*)	Survey Respondents	Response Rate (End-of-Semester Survey)
**P1**	Dr. T’s Tutor (ChatGPT)	Biochemistry (Metabolism)	Fall 2024	119	105/119	88.24%
**P1**	Dr. T’s Tutor (PlayLab)	Biochemistry (Metabolism)	Fall 2025	110	96/110	87.27%
**P1**	ANSwer Bot (PlayLab)	Physiology (Autonomic Nervous System)	Fall 2025	108	93/108	86.11%
**P3**	ZapNap Bot (PlayLab)	Neurology (Pain & Anesthesia)	Fall 2025	108	106/108	98.15%
**P3**	MindPath Bot (PlayLab)	Psychiatry (Schizophrenia, Delirium, Depression & Bipolar Disorder)	Spring 2025	108	106/108	98.15%
**P3**	MindPath Bot (PlayLab)	Psychiatry (Schizophrenia, Delirium, Depression & Bipolar Disorder)	Spring 2026	98	95/98	96.94%

Note: P1 = First-Year Pharmacy Students; P3 = Third-Year Pharmacy Students. All bots were deployed via PlayLab (PlayLab Inc., AI, Los Angeles, CA), except for Dr. T’s Tutor Fall 2024, which was deployed via ChatGPT (OpenAI). Survey respondents reflect standard end-of-semester course evaluations.

**Table 3:** Cohort Timeline.

Academic Year	Cohort	Instruction Mode	AI Tutor	Role in Study
**2023–24**	P1, P3	In-Person	None	Control
**2024–25**	P1, P3	In-Person	Dr. T’s Tutor (ChatGPT) MindPath (PlayLab)	Intervention Year 1
**2025–26**	P1, P3	In-Person	Dr. T’s Tutor (PlayLab) Answer bot (PlayLab) ZapNap (PlayLab) MindPath (PlayLab)	Intervention Year 2

**Table 4:** Normalized AI Tutor engagement data.

Tutor	Course	Duration	Sessions	Enrolled (*n*)	Sessions/Student/Week
**Dr. T’s**	Biochemistry`24	15 weeks	~300	~119	~0.17
**Dr. T’s**	Biochemistry`25	15 weeks	~240	~110	~0.15
**ANSwer**	Physiology`25	15 weeks	~90	~108	~0.06
**ZapNap**	Neurology`25	4 weeks	90	~108	~0.21
**MindPath**	Psychiatry`25	4 weeks	6	~108	~0.02
**MindPath**	Psychiatry`26	4 weeks	16	~98	~0.04

Notes: Sessions/Student/Week = Total Sessions ÷ [Enrolled (*n*) × Duration in weeks].

**Table 5:** AI tutor usage patterns for P1 vs. P3 students.

Student Level	Primary Use Pattern	Specific Query Examples	AI Tutor Response Strategy	Cognitive Load Theory Alignment
**P1**	Engagement & Retention	Requesting a step-by-step breakdown of metabolic pathways	Stepwise explanations with scaffolding	Reduces extraneous load through structured delivery
	Detailed Explanations	Asking for clarification on complex biochemical mechanisms	Detailed breakdowns with concept integration	Manages intrinsic load via chunking
	Relevant Examples	Seeking real-world applications of physiological concepts	Analogies and contextual examples	Enhances relevant load through meaningful connections
**P3**	High-Level Cognitive Synthesis	Differentiating complex disease presentations/assessment questions	Comparative analysis using analogies	Reduces cognitive load in complex differentiation tasks
	Nuanced Pharmacological Clarification	Clarifying nuanced concepts in anesthesia protocols and pain management	Stepwise explanations addressing conceptual bottlenecks	Manages intrinsic load for advanced clinical concepts

**Table 6:** Longitudinal AACP Standardized Proficiency Trends in P1 and P3 courses related to this study (Fall 2023–Spring 2026).

**Biochemistry—AACP Average Proficiency Trends (%)**				
**Metric**	**Fall 2023 (no AI)**	**Fall 2024 (ChatGPT)**	**Fall 2025 (PlayLab)**	
4300-05, 06 and 08	77.9	81.6	83.5	
Carb/AA/Lipid metabolism				
Metabolic Pathways/diabetes				
Vitamins & Minerals				
**Physiology—AACP Average Proficiency Trends (%)**				
**Metric**	**Fall 2023 (no AI)**	**Fall 2024 (no AI)**	**Fall 2025 (PlayLab)**	
4320-03	81.6	82.4	81.8	
Autonomic Nervous System				
**Neurology—AACP Average Proficiency Trends (%)**				
**Metric**	**Fall 2023 (no AI)**	**Fall 2024 (no AI)**	**Fall 2025 (PlayLab)**	
5335-01, 03	86.8	86.6	90.0	
Pathophysiology/Pharmacology				
**Psychiatry-AACP Average Proficiency Trends (%)**				
**Metric**	**Fall 2023 (no AI)**	**Fall 2024 (no AI)**	**Fall 2025 (PlayLab)**	**Fall 2026 (PlayLab)**
5368-01 and 03	86.3	86.2	85.8	88.8
Pathophysiology /Pharmacology				

**Table 7:** Preliminary themes from student comments regarding the reason to use the tutor.

Theme	Description	Example Student Language
**Positive feedback & overall satisfaction**	Students valued the additional help resource and had a very high satisfaction rate with the AI tutors.	“Her AI tutor, workbooks, and videos helped TREMENDOUSLY with learning the material.” “I really appreciate that Dr. T provides so many different resources” “I loved it. And also the Physiology:)”
**Convenient, on-demand support**	Students valued fast access during independent study, evenings, weekends, and late-night sessions.	“I was studying late at night and needed quick answers or testing myself.”
**Clarification of specific concepts**	Students used the tutor to resolve focused questions that did not necessarily require the professor’s input.	“I had small questions that I needed clarification on before I continued studying…”
**Concept integration across lectures**	Students used the tutor to connect distributed material across lectures and reduce the burden of searching through slides.	“I primarily used the tutor to connect the different lectures together rather than sifting through the hundred or so slides…”
**Self-testing and exam preparation**	Students requested practice questions, flashcard assistance, and review support.	“Example questions to give myself practice exams.”
**Reorganization of complex content**	Students used the tutor to reformat, simplify, or organize dense metabolic content.	“It helped fill the gap in my studying when the content was out of order or needed to be re-formatted/organized…”
**Variable adoption based on perceived need**	Some students found existing slides, games, or class materials sufficient and therefore did not use the tutor much.	“What we covered in class and what was available on the slides and from the games… were enough for my needs.”
**Platform/access** **barriers**	ChatGPT account creation, message limits, file upload limits, and potential cost-limited usage.	“I did not want to create a ChatGPT account…”; “The biggest issue… is that there is a limit to how many messages…”
**Trust and accuracy concerns**	Some students recognized the possibility of AI inaccuracies and wanted reliable, course-aligned responses.	“AI can also be inaccurate and provide false explanations.”

## Data Availability

The data presented in this study are available on request from the corresponding author and limited due to privacy restrictions related to student records.

## Supplementary Materials

The following supporting information can be downloaded at: https://www.mdpi.com/article/10.3390/pharmacy14070151/s1, File S1: Complete system protocol and detailed technical platform comparisons.
