# Supplementary material for: AI-Powered Personal Tutors as a Scalable Solution to the 2-Sigma Problem in Pharmacy Education
Source: Pharmacy (Basel). 2026 Oct 14;14(7):151. doi: 10.3390/pharmacy14070151 (PMC13709004; doi:10.3390/pharmacy14070151)
Supplement: Supplementary file 1 [file pharmacy-14-00151-s001.zip › pharmacy-4506393-supplementary.pdf]

Appendix A.

Platform Selection and Bot Development
All tutors were developed using a standardized, six-step iterative framework to ensure pedagogical consistency, following initial planning, model selection, customization/technical design, data preparation via Retrieval-Augmented Generation (RAG), implementation with validation and tuning, and final deployment. This process ensured that the instructional intent and content constraints remained equivalent across all tools, independent of the deployment platform.

Initial deployment [ChatGPT] - The pilot iteration, Dr. T's Tutor (Biochemistry, Fall 2024), was developed using the ChatGPT custom interface (Chat Generative Pre-trained Transformer, OpenAI©). This is a conversational, back-and-forth interface that can create new, original text responses because of its training on massive datasets of text from the internet. However, the bot's knowledge base was constrained to instructor-generated lecture transcripts and course materials using the platform's native file-upload feature. To maintain curricular fidelity and prevent the introduction of external, unverified, or unnecessary content, web browsing and image generation capabilities were intentionally disabled.

Model selection and Transition to PlayLab – Subsequent tutors, including the revised Dr. T's tutor (Fall 2025), ANSwerBot (Physiology, Spring 2025), ZapNap (Neurology, Fall 2025), and MindPath (Psychiatry, Spring 2025 and Spring 2026), were transitioned to PlayLab**.** This is an educational platform 501© nonprofit organization (Playlab Education Inc.), part of a global community dedicated to improving education, committed to creating scaffolded learning apps and "sandbox" environments for students to explore concepts safely. This shift was driven by three methodological requirements:

1) Accessibility – PlayLab removed the need for student subscriptions, eliminating a potential barrier to entry
2) Development efficiency – The platform provides an instructor-friendly interface for rapid content iteration and refinement.
3) Data collection – Critically, PlayLab offered an administrative dashboard with anonymized interaction logs. Unlike ChatGPT, which required students to manually generate and share conversation links via an anonymous Google Form, PlayLab allows for comprehensive qualitative analysis of all student-bot interactions while strictly preserving student privacy.

   The transition from ChatGPT to PlayLab represented an implementation refinement rather than a change in the educational intervention. Both platforms utilized Large Language Model (LLM) technology grounded in the same instructor-provided content via RAG. The core pedagogical objective (providing 24/7, curriculum-aligned, individualized support) remained identical across all study iterations.

Customization, Technical Design and Implementation - The technical design of the bots followed a Retrieval-Augmented framework (RAG, Li et al., 2025), implemented through the retrieval of instructor-created knowledge tools with semantically relevant content from a curated body of documents provided to condition the LLM. This approach allowed for the design of source-traceable responses grounded exclusively in instructor-created products, including lecture transcripts, video captions, and slide decks. To ensure curriculum alignment and prevent "hallucinations," the knowledge bases were strictly fenced within specific content domains. Web search and image generation capabilities were intentionally disabled to maintain a focused learning environment and prevent the introduction of extraneous or unverified

information from the broader web. Each AI tutor was governed by explicit system instructions to restrict its scope to the following areas:

- Biochemistry (Dr. T's Tutor): Limited exclusively to the metabolism of glucose, glucose homeostasis, complex carbohydrate metabolism, amino acids, and lipids.
- Physiology (ANSwer bot): Focused solely on the autonomic nervous system and associated visceral reflexes.
- Neurology (ZapNap): Restricted to the pathophysiology and pharmacology of pain and anesthesia.
- Psychiatry (MindPath): Focused on the pathophysiology and pharmacology of schizophrenia, delirium, depression, and bipolar disorder.

A primary design constraint for the P3 bots (ZapNap and MindPath) was the explicit prohibition of clinical diagnostic or treatment recommendations. System prompts were configured to have a pedagogical safety boundary and to redirect students to their clinical faculty for any queries involving patient cases or treatment decisions. This design ensured the tools functioned as foundational science tutors rather than clinical decision support systems, respecting the expertise of clinical instructors while providing 24/7 academic support.

To verify the efficacy of the established clinical fence, a robust testing process was conducted before deployment. The primary instructor performed adversarial testing by intentionally prompting the P3 bots (*ZapNap* and *MindPath*) with requests for specific drug dosages and treatment recommendations for hypothetical patient cases. In each instance, the bots correctly identified the clinical nature of the query, refused to provide a recommendation, and triggered the pre-configured system prompt, redirecting the user to clinical faculty (example: *"I can explain the mechanism of SSRIs, but for clinical dosing in a specific patient case, please consult your clinical instructor.").* This validation was repeated across several common clinical scenarios, achieving a 100% success rate in maintaining pedagogical boundaries and ensuring student safety.

The development of our AI tutors was guided by Cognitive Load Theory (CLT) and evidence-based principles for effective educational media, endorsed by AACP (Brame, 2017). To optimize the limited working memory of pharmacy students, the tutors were engineered to redistribute and shape mental effort across three distinct loads:

1. *Minimizing Extraneous Load*: By disabling web search and constraining the AI to specific course transcripts and videos, we prevented the introduction of irrelevant or conflicting information that could distract from the core learning objectives. This design stripped away avoidable cognitive friction, ensuring that working memory resources were not diverted to irrelevant content.
2. *Managing Intrinsic Load*: To handle the high element interactivity of complex metabolic, physiological pathways, and mechanisms of action, the tutors employed segmenting, breaking content into manageable chunks. The AI was also programmed to use signaling, highlighting important concepts through bold text (Brame, 2017). This matched task complexity to the learner's current state, allowing schemas to consolidate before more demanding problems were introduced or discussed.
3. *Optimizing Useful Load*: The tutors utilized conversational language, or the personalization principle (using "your" and "I"), to create a sense of social partnership between the student and the AI, which has been shown to increase student effort in making sense of the material (Brame, 2017). Furthermore, the bots were configured as Socratic partners rather than simple answer engines, prompting students to explain their reasoning or understanding, and engage in the effortful thinking required for durable schema construction.

Data Governance, Student Privacy, and Record Linkage Workflow

1. **Data Source Separation:**
   - *Aggregate Qualitative Logs:* Server transcripts generated via the PlayLab platform were accessed exclusively from the institutional administrative dashboard. The system did not collect, store, or export student names, email addresses, or IP addresses. All conversation threads were analyzed as de-identified aggregate text for query classification and thematic categorization.
   - *Individual Quantitative Linkage:* In the Fall 2024 pilot cohort (n =32), examination scores were linked to tutor usage via an IRB-approved post-examination voluntary survey.
2. **Linkage Mechanics & Bias Safeguards:**
   - Consenting students voluntarily submitted their institutional student identification numbers on the survey.
   - To prevent grading bias, the merge between survey responses and examination scores was performed exclusively by the primary investigator *after* all official course examination grades were locked and published to the registrar.
3. **Pseudonymization & Security Protocol:**
   - Immediately following the merge, institutional IDs were permanently removed and replaced with random study codes.
   - The de-identified research dataset was used for all statistical analyses.
